# Surgical Treatment of Exophthalmic Goiter1Read at the forty-third annual meeting of the Medical Association of Central New York, held at Syracuse, October 19 and 20, 1910.

**Published:** 1911-06

**Authors:** Martin B. Tinker

**Affiliations:** Ithaca, N. Y.


### Surgical Treatment of Exophthalmic Goiter[1]

By MARTIN B. TINKER, S.B., M.D.
Ithaca, N. Y.

SURGERY should be the last resort in the treatment of exophthalmic goiter as in many diseases. Conservative, conscientious surgeons all will assent to this. I begin with this statement in order that I may not be too greatly misunderstood when later I enlarge on surgical methods and results and insist on their importance. But by the last resort I do not mean that medical treatment should be continued for months and years until the patient is beyond all reasonable hope of relief. For some un-

---

1. Read at the forty-third annual meeting of the Medical Association of Central New York, held at Syracuse, October 19 and 20, 1910.

accountable reason our profession has been specially slow to accept surgery in the treatment of goiter. Many who recognise the value of surgery for simple goiter still consider exophthalmic goiter inoperable. Medical treatment is notoriously uncertain and inefficient in many of these cases. On the other hand Kocher, whose experience covers over twenty years with over 4,000 cases tells us that practically all goiters are curable surgically. If you doubt that distinguished internists share Kocher's opinion, take down your latest edition of Osler and read what he has to say about the treatment of exophthalmic goiter. An increasing number of medical men from Moebius, who wrote as early as 1895, have advocated surgery for exophthalmic goiter which proved intractable to medical treatment. When the public as well as the profession come to realise what surgery can do for goiter as well as they now understand the situation in appendicitis, much needless suffering will be spared, many will be able to do their share of the world's work who are now chronic invalids, and a good many unnecessary deaths will be prevented.

*Indications for Operation.*—Any considerable enlargement of the thyroid gland is not likely to yield to medical measures and is very likely, sooner or later, to give rise to serious trouble. Moderate enlargement is very often overlooked. Physicians who are not accustomed to see the exact conditions on the operating table will often say, "But there is no goiter in this case." It is not at all unusual to find the enlargement the size of an egg or larger in cases in which the attending physician feels that there is very slight enlargement. This is explained in several ways. Oftentimes the goiter is low in the neck so that under ordinary conditions half or more of it lies below or behind the clavicle and sternum. These intrathoracic goiters are much more dangerous than those situated higher in the neck. In other cases the gland on examination lies behind a thick sterno mastoid muscle. Nearly always physicians are surprised to see the size of the goiter when exposed on the operating table. I believe it is fair to say that enlargement the size of an egg or greater is an indication for removal in the vast majority of cases.

Difficulty in swallowing, especially if there are serious attacks of choking, is an urgent indication for operation. Reports of sudden death from choking come to me constantly and I have knowledge of a very large number of such cases. While many patients survive repeated attacks, others die, choked to death after a few warnings.

Any indication that the circulatory organs are suffering seriously also demands early operation. Doubtless the toxins in a case of hyperthyroidism may cause more or less myocarditis.

In any case the strain on the heart of prolonged tachycardia is great and whether weakness comes from muscle poisoning or from over exertion, a dilated heart is apt to result sooner or later. If operation is performed at a reasonably early date the heart usually returns to normal, but if too long delayed the impairment of this important vital organ may be permanent. The great extra burden carried by the circulation in such cases is often overlooked. Suppose the heart is beating at 120: if we consider the normal pulse rate 80, it is doing one-third more than usual work; 40 extra beats every minute; 240 every hour. If we estimate roughly that the energy required is equal to that of opening and closing the hand, and it is probably more, we could get some idea of the entire work put upon the heart by opening and closing the hand 240 times in an hour. Even a strong man would rapidly tire of this, and to keep it up for twenty-four hours would be impossible for most people, for it would mean 4,760 times in twenty-four hours. Besides the strain on the heart, the blood-vessels also suffer. Arteriosclerosis, in greater or less degree, is frequently found in people who have suffered from exophthalmic goiter for many years.

Persistent coughing and difficulty in breathing, which are not infrequently attributed by physicians unfamiliar with the disease to asthma or bronchitis, are occasionally seen. Several cases have come under my care in whom it was a permanent symptom. In such cases operation is indicated for the relief of the respiratory condition.

Extreme nervousness may also urgently indicate operative measures. In the advanced cases, organic change frequently takes place. In all well-developed cases of goiter, there is probably more or less mental disturbance and some of these patients seem on the verge of insanity but are relieved when the chronic poisoning is checked by removal of its source, the thyroid gland. Not all insanity found in goiter patients, of whom there are a large number in many of our state institutions, could have been prevented by timely removal of a portion of the thyroid, but if even a small percentage could have been prevented as seems likely, it would be a great gain. The tremor, sleeplessness and extreme restlessness are greatly benefited and the patients usually consider themselves cured after operation, even in the advanced cases, but it is doubtful if the damage to the nervous system is ever entirely repaired in such cases. Certainly a very prolonged rest cure is needed to bring about such a result. Of less vital importance is the bulging of the eyes. Exophthalmos, which has existed for a long time, is one of the most difficult symptoms to relieve. If the goiter is removed early, the eyes frequently return to normal, but improvement of the bulged eyes cannot be predicted even in fairly early cases with anything like the same certainty as relief of the extreme nervousness and rapid heart. In the more extreme cases the eyes are likely to suffer seriously because of exposure to dust, other foreign bodies and infection. In any case the deformity is a striking and disagreeable one and if it is to be prevented, early operation must be advised.

It is interesting to note that the symptoms of goiter are not unlike symptoms of some other forms of chronic poisoning. Tobacco poisoning is of striking similarity. The over use of tobacco gives a rapid, weak, tobacco heart. It causes the same general nervousness, sleeplessness and tremor. In certain cases there is impairment of vision. Any reasonable man would say at once that it would be highly illogical to treat tobacco poisoning by administering drugs without cutting out the use of the tobacco. It seems equally illogical to treat chronic poisoning from the over secretion of the thyroid by drugs without removing the cause of the poisoning.

*What Procedure Should be Advised.*—When we have decided that operation is indicated a still more important question is still left for decision. What is best to do in this particular case? How extensive an operation can safely be advised? This is best determined by a very thorough preliminary examination. In the general physical examination special attention should be devoted to the heart. In many cases it will be found greatly dilated as a result of the long continued over work. In such cases, even though no valvular lesion co-exists, the heart may be so weakened as to make immediate operation inadvisable. Functional trouble may also be marked. The heart's action is frequently very irregular and even intermittent. In certain doubtful cases a better idea will be obtained by asking the patient to walk up a flight of stairs. In others the irregularities will be only too evident without putting on any overstrain.

Even in young persons there is often thickening of the vessel walls and sclerosis. The blood pressure should be taken as well as the pulse rate and both should be charted at frequent intervals in doubtful cases. A high blood pressure is often seen in the acute toxic stage, abnormally low blood pressure when the vital forces are failing somewhat.

A careful blood examination, including hemoglobin estimation, red blood count and a differential count should be made in all doubtful cases and it is frequently helpful and instructive in cases in which there seems to be no doubt as to a safe blood condition. Anemia is common and rest in bed with feeding large quantities of freshly expressed beef juice from good round steak will usually improve the blood conditions very rapidly and bring the patient from a stage of blood impoverishment, in which operation would be accompanied by considerable risk, to a relatively safe procedure within a few weeks at the most. The differential count is characteristic in practically every case. A normal or somewhat low total number of leukocytes is accompanied by a relatively high number of mononuclear leukocytes. This lymphocytosis is of high diagnostic value in certain doubtful cases. When high, it indicates good power of reaction. When low, with a generally weakened condition, it sometimes indicates that the patient's vital forces are failing and that the wise course is to wait and see what can be done with general treatment before advising any operative measures. A greatly decreased coaguability of the blood is also usual in these cases. This is one reason why special care should be taken to secure all vessels which may bleed. Estimation of the coaguability of the blood is desirable if we are to carefully study all aspects of the disease and one of the various coagulo-viscometers may give valuable information in doubtful cases.

Albumen and some casts are almost always found in the urine if it is frequently examined. This need not necessarily contraindicate an operation, but if the condition is serious, it would indicate preliminary rest; if less grave, some less extensive operation. Sugar is occasionally present. The urinary condition almost invariably clears up satisfactorily after the toxemia is relieved by removal of a portion of the gland.

Surgical judgment as to what is best to do or leave undone is if possible even more important than skill in operating. Such judgment is based on experience from examination, operative treatment and post-operative care of a considerable number of cases as well as on the careful preliminary examination. The value of experience in estimating how much it is safe to do or whether it is wise to do anything at all in an operative way can hardly be over-estimated. Is preliminary treatment or immediate operation advisable? If it seems wise to operate, should we content ourselves with the ligation of a single artery, or can we safely remove a portion of the gland? In the more desperate cases, the operation can be divided into several stages.

*The Many Stage Operation.*—For the past three years, I have operated upon many desperate cases with ultimate satisfactory recovery. Many of these had hearts dilated to the axilla. At the beginning many were extremely anemic. Nearly all of these had considerable albumen and casts in the urine. In several, the heart's action was irregular, intermittent and feeble. Persistent cough and diarrhea were present with a few. All of these cases were treated by preliminary rest and building up. The operation itself was divided into many stages. In some of them: first, ligation of one artery on the least affected side of the gland; second, ligation of the artery on the side of greatest enlargement. Further treatment depended upon the improvement after preliminary rest and ligation. In some the gland could be removed at once. In others the gland was simply exposed and the wound packed with sterile gauze. In these cases of worst risk, the fourth stage consisted in simply removing the gland and again packing the wound with gauze, and at a fifth stage the wound was closed. The number of stages should be made as few as possible, but it is always better to keep within the limits of safety than to do too much. In all of the worst cases I have used local anesthesia in combination with the hyoscin morphine sleep. In two bad cases, occurring in young girls, nitrous oxide and oxygen anesthesia was used according to the rebreathing method of Gatch. In all cases the importance of a prolonged rest cure was urged upon the patient. They were given to understand that this was just as much a part of the treatment as the operation itself and unless they were willing to follow advice until a cure was obtained, operation has been refused.

*Immediate Results.*—First, the patient's condition. If the patient is in a fairly good physical condition, the risk to life is trivial. If he has been allowed to get into a very toxic condition, if the heart is dilated and its action irregular and intermittent, the danger may be considerable. The parallel between goiter and appendicitis may be fairly drawn. Patients taken early almost invariably recover. Patients neglected, in which temporising measures have been used until the condition has become desperate, frequently die in the case of either illness. The second factor is the surgeon. His experience, care and methods may easily make the difference between death and recovery. In no class of cases is experience and judgment more valuable. A thorough knowledge of the anatomy of the neck in its minutest detail is indispensable. Care is more important than speed in handling structures of vital importance. Kocher's methods, which have been less generally adopted than seems desirable, have given larger percentage of cures than any other reported up to date. In general, Kocher's methods include local anesthesia, painstaking dissection and careful arrest of hemorrhage. It may be safely said that in a case of moderate severity the operation for exophthalmic goiter is as safe as for any condition in surgery, in the hands of competent men. Tetany I have personally never seen and I believe its danger has been a good deal exaggerated. Kocher had seen only two cases in over 4,000 and none in his recent cases. Cachexia I have also never seen. It is always desirable to leave a much larger portion of gland than seems really needed as it is better to do a secondary operation to still further reduce the size of the gland than to deprive the patient of what is needed.

*Ultimate Results.*—Improvement is seen in almost every condition affecting the general health. The choking and difficulty in swallowing is relieved. The nausea and vomiting disappear. Diarrhea is immediately checked. Rapid increase in weight follows restoration to normal of the digestive tract, but is also influenced by improved metabolism which results from cutting out toxic poisons which have been absorbed in such large doses. The respiratory rate is reduced to normal and cough ceases. In one case a very persistent pleural effusion did not reaccumulate. The condition of the eyes is more difficult to influence than any other factor. When once the eyes have become badly bulged they seldom return to normal. If the patients are taken early, however, this trouble may be prevented: as in so many other conditions, prevention is much easier than cure. The nervousness improves rapidly in some, but in most cases much more slowly than many other symptoms. Tremor, nervous excitement and general unrest disappear in some cases only after a very prolonged rest cure. Probably the heart never entirely recovers if myocarditis has existed for a long time but in many a greatly dilated heart comes down to normal in a surprisingly short time. The quality of the pulse improves, the rate slows down to normal and the intermittent pulse is no longer noted. If the high tension and rapid action has not continued too long, arteriosclerosis is prevented. The amount of time required to give a permanent cure depends largely upon the condition of the patient before operation. Certain of the less advanced cases are also very obstinate in yielding to treatment. If proper operative measures are carried out and followed by prolonged rest cure, if directions are explicitly followed, I have yet to see a failure. In the less desperate cases, it would be safe to predict 99 per cent. of cures. I have now performed considerably over 100 consecutive operations without loss of life, taking the desperate with the relatively simple cases. This surely shows that the surgical treatment need not be extremely dangerous. That such results could be promised in another equally large series, including an equal number of bad risks would hardly be expected.

In conclusion, I would repeat what I have frequently said in discussing goiter: no class of patients are more grateful for the relief they receive. In no surgical condition is there a better prospect of permanent cure; no class of patients in my own experience so generally use their influence to send others to seek operative relief.

